# Public health education for midwives and midwifery students: a mixed methods study

**DOI:** 10.1186/1471-2393-12-142

**Published:** 2012-12-07

**Authors:** Jenny McNeill, Jackie Doran, Fiona Lynn, Gail Anderson, Fiona Alderdice

**Affiliations:** 1 School of Nursing & Midwifery, Queen’s University Belfast Medical Biology Centre, 97 Lisburn Road, Belfast, BT9 7BL, UK

**Keywords:** Public health, Midwifery, Education, Training

## Abstract

**Background:**

Current national and international maternity policy supports the importance of addressing public health goals and investing in early years. Health care providers for women during the reproductive and early postnatal period have the opportunity to encourage women to make choices that will impact positively on maternal and fetal health. Midwives are in a unique position, given the emphasis of the philosophy of midwifery care on building relationships and incorporating a holistic approach, to support women to make healthy choices with the aim of promoting health and preventing ill health. However, exploration of the educational preparation of midwives to facilitate public health interventions has been relatively limited. The aim of the study was to identify the scope of current midwifery pre registration educational provision in relation to public health and to explore the perspectives of midwives and midwifery students about the public health role of the midwife.

**Methods:**

This was a mixed methods study incorporating a survey of Higher Educational Institutions providing pre registration midwifery education across the UK and focus groups with midwifery students and registered midwives.

**Results:**

Twenty nine institutions (53% response) participated in the survey and nine focus groups were conducted (59 participants). Public health education was generally integrated into pre registration midwifery curricula as opposed to taught as a discrete subject. There was considerable variation in the provision of public health topics within midwifery curricula and the hours of teaching allocated to them. Focus group data indicated that it was consistently difficult for both midwifery students and midwives to articulate clearly their understanding and definition of public health in relation to midwifery.

**Conclusions:**

There is a unique opportunity to impact on maternal and infant health throughout the reproductive period; however the current approach to public health within midwifery education should be reviewed to capitalise on the role of the midwife in delivering public health interventions. It is clear that better understanding of midwifery public health roles and the visibility of public health within midwifery is required in order to maximise the potential contribution of midwives to achieving short and long term public health population goals.

## Background (5090)

UK policies [1-3] have increasingly recognised the importance of maximising health for infants and children at the start of life, and more recently since the Marmott review of health inequalities [4]. Internationally, there has been a similar focus in recent policy [5,6] and also seen in a report by the World Health Organisation [7]. Ensuring infants have a good start in life is at the cornerstone of good maternity care, as the origins of adult ill health have been linked with intrauterine fetal development, particularly size at birth, which is often referred to as the Barker Hypothesis [8,9]. Opportunities for all maternity care professionals exist to maximise both infant and maternal health throughout the perinatal period and address inequalities. However, midwives specifically have the potential to contribute significantly, given the centrality of building relationships with women within midwifery care [10] and the focus on promoting health [11,12].

Despite acknowledgement that public health is integral to midwifery [13,14] and a renewed emphasis on the contribution of maternity care to addressing health inequalities [15,16], key aspects of the public health role of the midwife have not been examined extensively in the research literature and, to date, limited attention has been given to how midwives recognise their contribution to public health. There are examples of midwifery led interventions, for example, weight management intervention [17], promoting maternal mental health [18] and innovative practice, such as, the partnership of the Royal College of Midwives (RCM) UK and Slimming World to support women [19]. However these are often not reported from a public health perspective and as a consequence, they may not be recognised by midwives as contributing to public health targets. In order to realise how midwives function as agents of public health and view their contribution to public health, it is important to explore the current education for midwives and how this prepares them for practice.

This study of midwifery education in relation to public health followed a review of midwifery practice across the UK [15], which involved conducting a systematic review of systematic reviews in relation to the public health role of the midwife [20-22]. A detailed study report was provided for the funders on completion of the study [23] which highlighted the need to clearly articulate the public health role of the midwife in education and in practice. The aim of this paper is to present the key findings relative to the scope of current educational provision in relation to public health and inequalities for pre registration midwives and, secondly, to explore the perspectives of midwives and midwifery students about public health.

## Methodology

The project comprised of two phases: a survey of all Higher Education Institutions (HEI’s) in the UK providing pre registration midwifery education, alongside focus groups with midwifery students and registered midwives across the UK. An Advisory Group (UK wide) was established and served to provide expert guidance on the project. Ethical approval was granted from the School of Nursing & Midwifery, Queen’s University Belfast Ethics Committee (Application Number: 0712010) and informed consent was sought from all participants.

### Phase 1

#### Design & sample

The aim of Phase 1 was to explore the current provision of public health education within pre registration midwifery curricula across the UK. A survey was constructed, which included both closed and open ended descriptive questions relating to the nature of public health education in pre registration midwifery curricula, with particular reference to topics, hours allocated, importance of public health to midwifery and gaps or limitations. Validated questions were used from a previous survey exploring public health education in Scotland and were piloted prior to commencing. The sample for Phase 1 was identified by contacting The Higher Education Statistics Agency (HESA) and searching the web pages of all UK HEI’S. A cross check was performed with the Nursing and Midwifery Council (NMC) Register of ‘Lead Midwife for Education’ (LME) database available on the NMC website (http://www.nmc-uk.org/Nurses-and-midwives/Midwifery/Midwifery-Education-and-Practice/Lead-Midwives-for-Education-LMEs) to ensure there were no omissions. Sixty HEIs in the UK were eligible (England: 46; NI: 2; Scotland: 8; Wales: 4).

#### Data collection & analysis

An invitation pack, including a letter of invitation, an information leaflet, a consent form and a copy of the survey, was posted to all identified institutions (n=60). Respondents had the option of completing the survey manually and returning in a prepaid envelope, completing it over the telephone with a member of the research team or completing it online via survey monkey. The project team also offered participants the option of completing the survey on the LME’s behalf by accessing the institutions’ curriculum documents. In this event, the survey was returned to the institution for approval before data analysis. Follow up telephone calls or emails were employed approximately 2 weeks later. Data collection commenced in January 2011 and was completed by April 2011. Data were entered initially to MS Excel and transferred to SPSS (Version 18) for analysis. Basic descriptive statistics were conducted and data from open ended questions were categorised thematically.

### Phase 2

#### Design &sample

In Phase 2 the aim was to conduct focus groups across England, Scotland, NI and Wales with midwives and with midwifery students to ascertain their perspectives on how education around public health and inequalities relates to practice and service delivery. The samples for the focus groups were recruited from the Royal College of Midwives (RCM), UK and selected institutions providing midwifery education. The RCM facilitate regular meetings with the country specific Boards (England, NI, Scotland and Wales) including, for example, Clinical Leads, Heads of Midwifery (academic and clinical), Consultant Midwives and Supervisors of Midwives. The RCM offices in England, Scotland, Wales and NI were contacted to arrange the focus groups and distribute the email invitation. The LME in each of the selected institutions was contacted to introduce the study and invite midwifery students to participate.

#### Data collection & analysis

The focus groups (conducted by JD, FL, GA & JM) were audio recorded and transcribed into MS Word independently. A schedule was designed to be used as a loose topic guide and to act as a prompt if required. Data collection commenced in January 2011 and was completed by April 2011. The transcripts were analysed by content primarily by JM with input from all members of the project team regarding emerging categories. Content analysis involves the identification of key topics or categories within the transcripts and then looking for relationships within the categories [24].

## Results

### Phase 1

Of the sixty institutions identified, 55 were eligible as they currently offered pre registration midwifery education. A total of 29 institutions responded (53%) in relation to 37 programmes (3 year and 18mth programmes), of which 23 were in England, 3 in Scotland, 2 in Wales and 1 in NI. Participants responded in a variety of methods: 15 (52%) replied via survey monkey: 10 (35%) via post: 3 (10%) sent their curriculum documents for completion by the project team and 1 (3%) completed over the phone.

#### Explicit reference to public health in midwifery curricula

**Figure 1:**
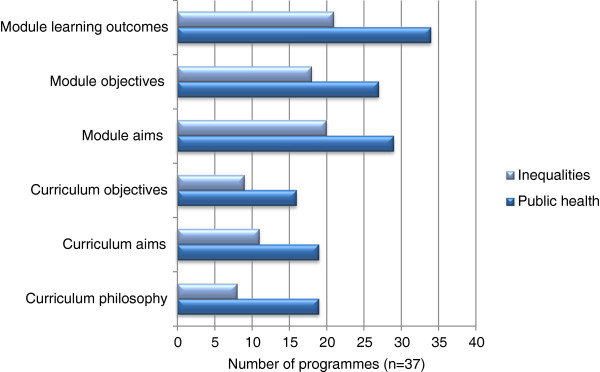
Explicit Inclusion of public health/inequalities in pre registration programme documentation.

The pre registration survey asked respondents to state how explicit (direct reference) the inclusion of public health was in the curriculum philosophy or programme and module aims/objectives. The results are presented in Figure 1.

#### Public health topics included

**Figure 2:**
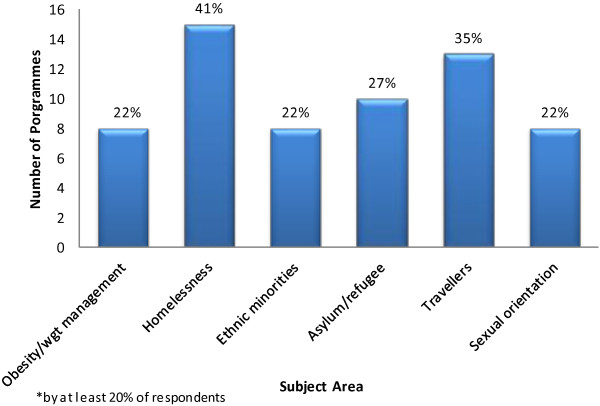
Specific public health and inequality subject areas not offered* pre registration programmes.

Respondents were invited to select from a list of pre defined topics on public health and inequalities and indicate whether they were included in their provision of pre registration education for midwives (Table 1). Participants were invited to indicate the approximate number of hours allocated to the list of topics. Table 1 demonstrates the considerable variation across institutions both in relation to the topics provided and the hours allocated, for example, three institutions stated they did not cover the principles of public health, five reported they did not include epidemiology and the number of hours allocated to perinatal mental health ranged from 1.5 to 14. A number of respondents also reported that several subject areas were not offered, as illustrated in Figure 2.

**Table 1:** *Pre registration public health and inequalities subject areas*

	**Method of inclusion**				**Length of time of session (s)(range in hours)**
	**Integrated**	**Specific**	**Not included**	**Missing**	
**Principles of Public Health**	22	11	3	1	2-10
**Health & Social Care Policy**	29	5	2	1	2-6
**Epidemiology**	26	2	5	4	1.5-6
**Substance Misuse**	30	3	1	3	1.5-11
**Smoking**	30	3	2	2	1-6
**Obesity/Weight Management**	25	2	8	2	1-6
**Maternal Nutrition**	31	0	4	2	1.5-3
**Health Promotion**	27	7	1	2	1.5-4
**Health Education**	28	5	2	2	1.5-4
**Blood Borne Viruses**	31	2	3	2	1-4
**Domestic Violence**	33	1	1	2	2-6
**Homelessness**	19	0	15	3	1.5-3
**Ethnic Minority**	25	2	8	2	1.5-4
**Asylum/Refugee**	23	2	10	2	1-6
**Travellers**	21	0	13	3	1.5-3
**Parent Support Initiatives**	29	2	4	2	1-6
**Breast Feeding/Infant nutrition**	31	4	0	2	3-16.5**
**Teenage Pregnancy**	28	2	4	3	1-6
**Sexual Health**	28	3	3	3	2-10
**Sexual Orientation**	25	2	8	2	1-6
**Perinatal Morbidity**	30	3	2	2	2-12
**Perinatal Mental Health**	31	4	0	2	1.5-14
**Child Protection**	33	1	0	3	1-6
Optimising Birth*	19	2	0	16	2.5-7

*paper responses only.

**range excludes 1 institution which offered 140 hours.

#### Curriculum gaps and limitations

Respondents were asked to identify any gaps or limitations in the current provision of public health education. Twenty five (68%) respondents reported there were no gaps, six (16%) reported they felt there were gaps; and six (16%) did not respond. There was recognition that public health was explicit in institutional programmes, however, it was also reported that more time was needed to explore theoretical models and often learning was solely focused on practical aspects. Some respondents who reported that they felt there were no gaps in the curriculum also commented that the public health elements of their undergraduate curricula depended on good links with practice for example the facilitation of clinical placements which provide exposure to public health roles. It was highlighted that the curriculum needed to be regularly revisited in order to ensure relevance. Specific topic areas where gaps were identified by respondents included perinatal mental health, asylum seekers and homelessness, obesity, nutrition and alcohol.

#### Public health as core to midwifery

Respondents were asked to rate on a scale of 1–5 (5=essential) how much they thought public health was part of the core role of the midwife. All participants denoted a score of 4 or 5 with the exception of one, indicating the majority considered public health as an essential element of core midwifery practice.

### Phase 2

Nine focus groups with 59 participants (34 midwifery students and 25 registered midwives) were conducted. Four focus groups with students were conducted in three participating institutions (England, NI and Scotland) and five focus groups were held with registered midwives; participants included managers, midwives from practice, public health specialists and educationalists in England, NI, Scotland and Wales. Data from the focus groups are presented in relation to three key themes: understanding public health in midwifery; the reality of practice; knowledge and confidence about public health.

#### Understanding public health in midwifery

Throughout the group discussions it was evident that midwifery students did not have clear understanding of the public health role of midwives. In some groups, initially it was seen as a specialist area and not as core, given that midwives cannot be ‘experts’ in all areas. However, as the discussions continued within groups, there eventually (and usually) was consensus that public health was integral to midwifery practice and input from multidisciplinary teams or specialists could be utilised for additional support.

“I think the role of the midwife is really important but when I was doing my bit of research for my assignment one of the key things that was out there, a lot of midwives don’t accept that they have a role in public health” (Scotland Student Group)

In all of the focus groups with registered midwives the definition of public health relative to midwifery was difficult to pinpoint precisely and generally the question was met by initial silence. One group identified that it was important for midwives to have ‘their’ definition of public health and what it means in midwifery practice as other disciplines have a clearer understanding of what public health is.

“So I think what midwives need to do is (consider) what is our meaning, our understanding, our domain, what is our package of public health? What do we mean by it? What would be our targets? What would we want to see as perhaps, we can’t control the whole population but we can look at the whole of childbirth, say from maybe a little bit of preconception right up to is it midwives’ role up to 28 days after birth? What kind of targets, goals, public health things would fit in?” (England Midwifery Group)

Discussions with registered midwives were generally consensual about public health as an aspect of midwifery practice, although, there was often debate as to the extent of this role and boundaries regarding core or specialist practice. Terminology, such as ‘crucial’, ‘pivotal’, ‘the foundation of it’, ‘significant role’, was used to describe the public health role of the midwife in relation to the core aspect, although, within groups there was confusion relating to if and how midwives viewed themselves as public health practitioners.

‘It’s got to be the core function and then we build on top of that’ (Wales Midwifery Group)

One group discussed how difficult it was to marry the goals of public health and the aim of holistic midwifery care. It was proposed that the goals of public health are overarching and at population level, whereas in midwifery care the aim is more towards an individualised approach tailored to the specific needs of women and their families, and therefore, this may result in conflict (see quote below). This was not discussed voluntarily in subsequent groups, however, the moderator of the final focus group introduced the idea and the concept was generally agreed.

“.....public health tends to take a very global approach and they want everybody vaccinated and everybody to give up smoking and everybody to breast feed. And the reality is that midwives, we’re actually dealing with individuals who are giving us very good reason for why they’re going to continue smoking and why they’re not breastfeeding which may not fit with the public health agenda. I think that there’s a fundamental problem between imposing that perhaps, on a midwife who is actually working with an individual and understands that woman’s context. Yes, she knows it’s not good for her to smoke. Yes, she knows it’s going to give her cancer or whatever in the long term but right now she’s just trying to survive. And I think trying to superimpose this public health practitioner role on a midwife could actually lead to role confusion or completely role rejection”. (Scotland Midwifery Group)

#### The reality of practice

A general lack of confidence and some anxiety around discussing specific public health related topics with women was reported by midwifery students at various stages of their training e.g. smoking cessation.

“I’ve completely avoided that huge area of public health and midwifery and I feel terrified of it now, you know, if I were to get a woman who was saying, ‘I’m smoking, what can I do about it’... I wouldn’t know”. (England Student Group)

Students were also aware of the impact of busy clinical environments and the subsequent effect on the ability of midwives to address or discuss public health issues.

“I think time’s a big issue with all public health. I think midwives don’t have enough time to deal with all the public health issues that they need to deal with” (NI Student Group)

Although it was generally recognised that public health interventions and addressing inequalities are part of the midwives’ role, barriers in clinical practice were identified as influential on the effectiveness of that role. Barriers discussed included the shortage of time available clinically to care for women, the difficulty of providing copious health promotion messages at the booking interview, the ‘tick box’ approach to care, midwives’ reluctance to develop conversations with women due to a lack of time, continual ‘adding onto’ the midwives’ role, models of care and the lack of vision regarding long term outcomes of care. Additional barriers were identified that focused more generally around professional issues, such as, heavy administration and bureaucracy, work load volume and leadership. However, despite the recognised barriers, groups were unanimous that pregnancy was a time of opportunity for midwives to promote the overarching goals of public health. The recognition of pregnancy as a time of ‘opportunity’ was resonant through all the focus groups and there was unanimous agreement both within and between groups that pregnancy is a time in women’s lives which could be influenced with regard to a public health message.

“You know, I think what we do have as midwives is a captive audience. We have an opportunity. We engage with women, somewhere in and around six to twelve weeks in their pregnancy depending on how early they do their pregnancy test and who they contact first. And we have access to those women who are like sponges for information for at least six months and it is an opportunity” (NI Midwifery Group)

#### Knowledge and confidence about public health

The majority of students were able to discuss key public health topics relevant to midwifery practice and perceived their level of theoretical knowledge was good; however they reported that practical delivery was difficult. Several groups suggested some additional solutions, such as, motivational interviewing or training in communication skills through role play as highlighted below.

“Participant 1: But it’s hard, I think, for us I think to go out and start telling people this. I think you need more than a, confidence lessons or something...

Participant 2: Or, just different approaches to how you go about health promotion. You know, do you ask how, what the woman knows about it first and getting into like dialogue and conversation as opposed to telling the woman what to do.

Participant 1: Yeah...yeah, so like more of the ‘how to’.

Participant 2: Yeah, definitely. Role play....I think that would be really good” (NI Student Group)

Barriers to increasing knowledge were identified by the focus groups with registered midwives. These related to the availability of training, difficulty releasing staff for training and the type of training that is needed. The majority of groups acknowledged that training exists, however, the topic is often politically motivated or a current hot topic, for example, the focus on obesity and weight management during pregnancy. Another issue raised was the availability of funding for training; funding was prioritised for courses where the aim was to develop skills of direct benefit to practice i.e. medical prescribing or examination of the newborn skills over developing theoretical knowledge, as illustrated by a quote from a NHS midwifery manager, below:

“If a midwife came to me and said I want to go and do a module at (a HEI) or wherever on public health, unless she was doing it as part of a degree I can’t see her coming forward to do it, and I couldn’t support her unless I had a particular role for her” (NI Midwifery Group)

There was a recognition that public health was more prominent on pre registration education curricula and that newly qualified midwives were perceived to be ‘steeped in public health” (Scotland) and ‘more conscious of public health than midwives trained a few years back’ (Wales). However other groups felt that while this may be true, there were concerns around the general lack of midwives’ confidence to discuss many public health issues with women, for example obesity, weight management, and routine enquiry about domestic abuse.

Some of the discussion in the focus groups (registered midwives) outlined potential measures to address the barriers in order to maximise the public health role of the midwife. Recognition of the need for more training was identified and several examples of innovative practice were provided. For example, a NHS service manager gave an example of how funding had been obtained through the British Heart Foundation for a midwife to link into a community based obesity networking and motivational programme.

Several methods of training to address gaps in the effectiveness of a midwifery public health role were suggested. Online training in the form of a toolkit was suggested in one group. This would have the advantage that midwives could access it in their own time. However, another group felt that online learning was problematic in the area of public health, as there was a need for an interactive element and also monitoring compliance with online learning could be difficult if the training was not mandatory. Increased knowledge of interventions that midwives could conduct was discussed as something that would be helpful. Brief intervention training, which has been used effectively in other areas of practice, was also raised as a potential for midwives in the area of public health. Underlying the recognition of training, however, was the need for more emphasis on the application of public health to midwifery and for all midwives to understand better the relationship between public health and midwifery.

“.....so I think the longer term thing would be to change the culture of how midwives see their role in public health and accept that and maybe see that it’s not an add-on to our role” (NI Midwifery Group)

“I think a lot of it too is, [that] you do have to get underneath the midwife’s thought processes as well, in it all..if they’re going to deliver the positive message you’ve got to understand them, haven’t you, as a person and build their confidence” (Wales Midwifery Group)

## Discussion

Following analysis of the results from Phase 1 and Phase 2, the findings were further considered comparatively in relation to the key themes emerging from each phase. This process resulted in identification of three clear issues which will require significant consideration from the perspective of policy makers, education providers, midwifery researchers and midwives in practice in order to maximise the public health role of the midwife moving forward. The themes are further outlined in the following paragraphs under broad headings: understanding the public health role of the midwife; visibility of public health in midwifery and the direction of public health education in midwifery.

### Understanding the public health role of the midwife

It was consistently difficult for both midwifery students and registered midwives to articulate clearly their understanding and definition of public health in relation to midwifery. This lack of clarity created confusion around terminology in relation to public health and the subsequent application of the concept of public health in everyday midwifery practice. This was a similar finding to research [25] which explored perceptions of health promotion with midwifery students and reported a limited understanding of health promotion in the context of public health and lack of clarity around health promotion in midwifery practice, although, the sample size was small (n=8). In order to promote the public health role of midwives, further training in relation to public health awareness and how it relates to core midwifery practice will need to occur before any real progress can be made [26]. Within the focus groups it was clear that some midwives and the majority of midwifery students did not view themselves as public health practitioners or would not have described much of core midwifery practice as public health and, yet, the survey indicated that nearly all HEIs viewed public health as core to midwifery. The dissonance between the perspectives of educational providers and midwifery students and midwives is important to note and may explain some of the challenges reported when discussing public health topics in practice. To ensure the midwifery contribution to public health goals is valued it is vital that midwives and midwifery students recognise that much of what they do falls under the banner of public health and as such is acknowledged primarily by the midwifery profession but also other disciplines.

### Visibility of public health in midwifery

The current pre registration curriculum refers to essential competencies which must be achieved in order to register with the NMC. The concept of public health is evident and underpins many of the requirements, for example students are required to ‘actively encourage women to think about their own health and the health of their babies and families, and how this can be improved’. The term public health is only explicitly used once: ‘planning and offering midwifery care within the context of public health policies (p26)’ [27]. Pre registration education in relation to public health is for the most part integrated into the curriculum with very few universities offering specific modules. Whilst this was acknowledged in the focus groups, as parallel to how midwifery and public health are related i.e. it underpins all of what midwives do, this integrated approach potentially raises concern if linked to the lack of clear definitions and value of the public health role of the midwife. The intrinsic embedded nature, whilst philosophically sound, may contribute to the lack of recognition or awareness about public health within midwifery.

This highlights a major challenge in relation to public health and midwifery and suggests that future work in midwifery education and practice must focus on promoting a clear, visible public health role as core to midwifery [28]. One of the key recommendations of the Public Health Midwifery 2020 Work Stream Report [28] indicated that midwives need to capitalise on the opportunity to deliver evidence based public health interventions. Although a systematic review of reviews on the public health role of the midwife [20] identified several midwifery interventions from review evidence that midwives could implement, generally, the evidence was very limited. A subsequent review [22] reporting on high quality effective interventions identified a clear need for further research in this area suggesting that the provision of pre registration midwifery education in relation to public health needs to be reviewed in order for midwives to have a clearer understanding of their public health role and subsequently evaluate their practice.

### Direction of public health education in midwifery

Findings from the current study indicate there is reasonable consistency across the UK in terms of provision of pre registration education, which is to be expected given the NMC requirements for entry to the register as a midwife. Although the major topics are covered by the majority of HEI’s there was some variation in the provision of education for current hot topics e.g. obesity and weight management or maternal nutrition. This may reflect the time lag between what is current and the necessary administration around changing curriculum to meet NMC requirements or that pre registration curriculum are generally only renewed every 3–5 years However, this raises questions about the decision and rationale for inclusion of core and specialist topics. The role of specialist practitioners was referred to in the focus groups and this may be the mechanism to address the delay in translating current topics of interest in educational curricula. For example specialist practitioners could be routinely invited to present a guest lecture for midwifery students thereby increasing exposure to current work and relevant good practice. Brief Intervention Training, based on the principles of motivational interviewing to improve communication [29], was also suggested as a possible solution to improving training for midwives in relation to public health. This type of intervention has been reported previously as having potential to improve counselling by midwives on smoking cessation, as the observed communication styles (traditional, authoritarian and paternalistic) were not effective [30].

The Education Workstream Report from Midwifery 2020 [26] specifically noted ‘that knowledge and skills regarding public health and well-being need to be appropriately strengthened within pre registration programmes’ (p13). Public health education in relation to midwifery, which focuses more specifically on the public health role of the midwife rather than the current model, where the midwife is regarded as an agent who delivers health education or promotion messages, could potentially address some of the difficulties highlighted in this study. In addition it is important that public health is not seen as an added extra for midwives but as core to the philosophy of care [31]. Emphasising the public health role of the midwife would enable a better fit with the provision of midwifery care within a social model, taking into account the context in which health promotion or health education is delivered. Such training would enable midwives to visualise and apply the concept of public health to midwifery practice and improve their overall understanding of public health. Subsequent provision of care would then be framed in the context of impacting on long term health outcomes of the broader population [32].

### Strengths and limitations

Designing the questionnaire for Phase 1 was challenging, as the aim was to collect relevant detailed data whilst balancing this with the completion time. The nature of the questionnaire required respondents to refer to curriculum document(s) and, therefore, was time consuming to complete. To ensure respondents received maximum support throughout the process, the researchers kept in close email and telephone contact. In addition, we attempted to maximise the return rate by providing various options for completing the questionnaire, including an online option and completion by the researchers through the use of relevant curriculum documents and/or telephone. Telephone and email reminders were also used to increase the response rate. In Phase 2 the focus groups were generally representative of both students and practitioners due to the variety of years of experience/education, gender as appropriate to a midwifery profile and current employment, although, some groups had small numbers. One country within the UK was not represented in the student focus groups and, therefore, may have provided additional perspectives had the time frame permitted approaching other institutions. The findings from this study may be limited to a UK setting; however, it could be easily replicated in other countries.

## Conclusion

It is clear from this study that the current approach to public health education within pre registration midwifery should be reviewed in order to facilitate better understanding of midwifery public health roles and, therefore, maximise the visibility and potential contribution of midwives to achieving both short and longer term public health population goals. It is also essential for registered midwives to have a clear understanding of their public health role in order to implement and evaluate interventions and provide evidence based care. The findings from this study suggest that future research needs to explore mechanisms that would facilitate improved understanding by midwives of their contribution to public health and translation of knowledge into practice. The contribution of midwifery to public health has been relatively underplayed and, as the drive to meeting targets focusing on improving population health and reducing inequalities intensifies (particularly for children at the start of their lives), it is timely for midwives to recognise and assert the potential of their contribution.

## Competing interests

The author (s) declare they have no completing interests.

## Authors’ contributions

All authors (JM, JD, FL, GA and FA) contributed to the conception and design of the study, data collection, analysis and interpretation; JM prepared the first draft of the manuscript and all other authors provided guidance and critical revisions. All authors approved the final version for submission.

## Pre-publication history

The pre-publication history for this paper can be accessed here:

http://www.biomedcentral.com/1471-2393/12/142/prepub
